# Community engagement and population coverage in mass anti-malarial administrations: a systematic literature review

**DOI:** 10.1186/s12936-016-1593-y

**Published:** 2016-11-02

**Authors:** Bipin Adhikari, Nicola James, Gretchen Newby, Lorenz von Seidlein, Nicholas J. White, Nicholas P. J. Day, Arjen M. Dondorp, Christopher Pell, Phaik Yeong Cheah

**Affiliations:** 1 Mahidol-Oxford Tropical Medicine Research Unit, Faculty of Tropical Medicine, Mahidol University, Bangkok, Thailand; 2 The Malaria Elimination Initiative, Global Health Group, University of California, San Francisco, CA USA; 3 The Ethox Centre, Nuffield Department of Population Health, University of Oxford, Oxford, UK; 4 Centre for Tropical Medicine and Global Health, Nuffield Department of Medicine, Churchill Hospital, Oxford, UK; 5 Centre for Social Science and Global Health, University of Amsterdam, Amsterdam, The Netherlands

**Keywords:** Malaria, MDA, Community, Engagement, Population coverage

## Abstract

**Background:**

Mass anti-malarial administration has been proposed as a key component of the malaria elimination strategy in South East Asia. The success of this approach depends on the local malaria epidemiology, nature of the anti-malarial regimen and population coverage. Community engagement is used to promote population coverage but little research has systematically analysed its impact. This systematic review examines population coverage and community engagement in programmes of mass anti-malarial drug administration.

**Methods:**

This review builds on a previous review that identified 3049 articles describing mass anti-malarial administrations published between 1913 and 2011. Further search and application of a set of criteria conducted in the current review resulted in 51 articles that were retained for analysis. These 51 papers described the population coverage and/or community engagement in mass anti-malarial administrations. Population coverage was quantitatively assessed and a thematic analysis was conducted on the community engagement activities.

**Results:**

The studies were conducted in 26 countries: in diverse healthcare and social contexts where various anti-malarial regimens under varied study designs were administered. Twenty-eight articles reported only population coverage; 12 described only community engagement activities; and 11 community engagement and population coverage. Average population coverage was 83% but methods of calculating coverage were frequently unclear or inconsistent. Community engagement activities included providing health education and incentives, using community structures (e.g. existing hierarchies or health infrastructure), mobilizing human resources, and collaborating with government at some level (e.g. ministries of health). Community engagement was often a process involving various activities throughout the duration of the intervention.

**Conclusion:**

The mean population coverage was over 80% but incomplete reporting of calculation methods limits conclusions and comparisons between studies. Various community engagement activities and approaches were described, but many articles contained limited or no details. Other factors relevant to population coverage, such as the social, cultural and study context were scarcely reported. Further research is needed to understand the factors that influence population coverage and adherence in mass anti-malarial administrations and the role community engagement activities and approaches play in satisfactory participation.

**Electronic supplementary material:**

The online version of this article (doi:10.1186/s12936-016-1593-y) contains supplementary material, which is available to authorized users.

## Background

Malaria remains a leading global health concern and although, in South East Asia, malaria-related morbidity and mortality has seen recent declines, the spread of drug resistant *Plasmodium falciparum* parasites poses serious challenges to prevention and control efforts [1]. If left uncontained, the likely spread of artemisinin-resistant *P. falciparum* from Asia to Africa would have a catastrophic impact in the region where malaria-related mortality is highest [2].

This scenario has prompted urgent efforts to eliminate falciparum malaria in South East Asia [3, 4]. One approach, targeted malaria elimination (TME), combines conventional malaria prevention and control activities (reinforcing the network of village malaria workers (VMWs) to deliver appropriate case management and distribute long-lasting insecticide-treated bed nets (LLINs), with the mass administration of an artemisinin combination therapy. The mass drug administration (MDA) component of TME entails delivering a curative anti-malarial dose to all individuals within a community, irrespective of malaria infection status, to interrupt local transmission [5].

**Fig. 1:**
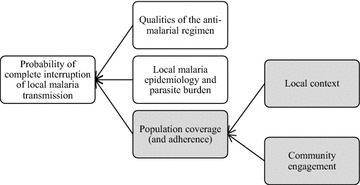
Factors affecting the probability of completely interrupting local malaria transmission through mass antimalarial administration. Highlighted in* grey* are areas of concern for this review

Over the past century, MDA has been used as a strategy for malaria control with varying degree of success [6]. Past mass anti-malarial administrations, which often entailed sub-therapeutic doses, have been blamed for accelerating drug resistance [5, 7]. This is less likely within TME because therapeutic doses of combination anti-malarials are administered [5–8]. Interrupting local transmission through MDA is dependent on several factors, particularly the local malaria epidemiology, the characteristics of the anti-malarial regimen, and population coverage and adherence (Fig. 1). In recent years, modelling studies have been helpful in determining the level of coverage required in specific epidemiological situations to interrupt transmission [9]. The level of coverage required to ensure the effectiveness of the MDA and how to define coverage is debatable particularly with regard to specifying numerator and denominator. For instance, many MDA studies, exclude pregnant women and young children because of concerns of toxicity. It is clear that the local social context and the community engagement activities that accompany the MDA influence coverage and adherence [9–17].

Community engagement is variously defined in the global health literature [18]. Some scholars emphasize community engagement as promoting ethical conduct of research, whereas other definitions focus on ‘working collaboratively’ with communities ‘to address issues affecting the well-being of those people’ [18, 19]. With regard to programmes of MDA, community engagement entails a range of activities—for example, employing community members and providing health education—that often focus on promoting population coverage and adherence [14, 19–22]. Although recognized as influencing coverage, community engagement is often scarcely reported and, to date, little effort have been made to analyse the impact of specific community engagement activities or approaches on MDA coverage.

With a view to designing community engagement activities for TME across the Greater Mekong sub-region, a systematic review of community engagement and population coverage within mass anti-malarial administrations was conducted [6, 23]. This article examines: (1) population coverage, (2) community engagement activities and (3) the relationship between (1) and (2) in previous mass anti-malarial administrations.

## Methods

This review builds upon an earlier Cochrane review of mass anti-malarial administrations by Poirot et al. [23]. For the Cochrane review, the following databases were searched: Cochrane infectious diseases, Group Specialized Register, Cochrane central register of controlled trials (CENTRAL), Cochrane Library, MEDLINE+ , EMBASE, CABS Abstracts and LILACS (Additional file 1). Search terms were: A. Anti-malarials: exp Antimalarials or exp malaria/or antimalarial* or anti-malarial* or schizonticidal* or gametocidal* or hypnozoiticidal* or drug* or treatment* or malaria* and B. Mass Administration: mass or coordinate* or administ* or distribut* or applicat* or use or therap* or treatment*.

**Fig. 2:**
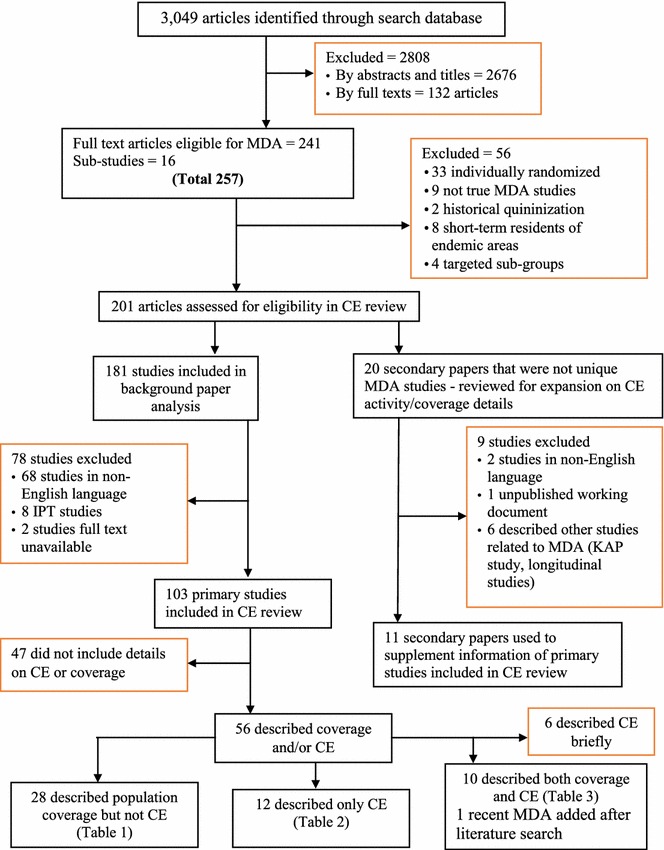
Assembly of the reviewed literature

From these searches, 3049 articles were retrieved and reviewed (Fig. 2). Of these articles, 241 were selected based on the eligibility for MDA [23]. Further review by Newby et al. [6] identified 16 sub-studies that resulted in a total of 257 full text articles. These 257 articles were subjected to the exclusion criteria (Table 1). This resulted in 201 articles published between November 30, 1931 and August 24, 2011 that were assessed for inclusion in the current review. To examine the maximum number of studies that might have documented community engagement activities and population coverage, articles excluded from previous reviews were included: articles that reported MDA for sub-groups rather than entire populations, described the delivery of inadequate treatment doses, documented the mass screen and treat approach and those that were not unique MDA studies (e.g. multiple publications documenting different components of the same study).

**Table 1:** Inclusion and exclusion criteria for review

Inclusion criteria to assess eligibility for review:	
1. All randomized and non-randomized studies of mass administration of anti-malarials, including cluster-randomized trials, non-randomized controlled studies and uncontrolled before-and-after studies that measured at least one outcome of interest in the target population	
2. Additional: inclusion of secondary papers related to MDA	
Exclusion criteria to assess eligibility for review:	
1. Individually randomized studies	
2. Studies in which the primary focus was not MDA (e.g. historical program reviews that merely made mention of MDA activities, community surveys done in conjunction with MDA)	
3. Studies using an indirect approach to MDA, where anti-malarials are added to essential foodstuffs, usually dietary salt	
4. Historical accounts of mass quinine distribution in the early 20th century	
5. Studies targeting short-term residents of malaria endemic areas (e.g. military, laborers)	
6. Studies written in languages other than English	
7. All intermittent preventive treatment (IPT) studies (e.g. delivering treatment to infants and young children, treating malaria-related febrile illness, home based treatments and comparison of delivery methods; factors that would greatly bias the choice of participation)	

Two authors (BA and NJ) independently retrieved and reviewed the articles. Firstly, all the literature pertaining to malaria-related MDAs was reviewed to extract population coverage data. In this review, population coverage has been defined as the total proportion of the target population who took anti-malarials with or without directly observed treatment (DOT) during the entire course of the MDA. Secondly, the articles were sorted according to the reports of community engagement activities. Thirdly, a thematic analysis of the literature that addressed the population coverage and/or community engagement was conducted.

Of the 201 articles, 181 reported primary studies and 20 reported secondary studies, which gave additional details on the primary studies. Seventy-eight of the 181 primary studies were excluded. These were 68 non English articles, eight that reported intermittent preventive treatment (IPT) and two of which the full text were not available resulting in 103 studies. Among these 20 secondary studies, nine were excluded: two were in languages other than English, one was an unpublished working document and six described studies indirectly related to MDA (such as knowledge, attitude and practice studies, and longitudinal studies), resulting in eleven secondary studies. Of these 11 studies, two were included in the previous review of community participation by Atkinson et al. [20]. These 11 articles were reviewed for their additional details on community engagement and coverage and were used to supplement the information detailed in Tables 2, 3 and 4.

**Table 2:** Studies documenting population coverage only (n = 28, in chronological order)

Author, year, country	Epidemiology (baseline parasitemia)	Study type and the context	Anti-malarial	Coverage/%	Additional interventions
Kingsbury 1931, Malaysia [58]	28% (Malaria incidence had been high for many years in the rubber estate)	Non-RCT Plasmoquine was administered to all individuals living in rubber plantation and the control population was chosen from another rubber plantation estate. The population composed of labourers working in rubber industry and the considerable fluctuation of population was observed over the years	Plasmoquine	97	Larviciding
Kligler 1931, Palestine [59]	69.4% in children age 0–14 years old	Before and after study carried out in 5 villages adjacent to Huleh marsh	Plasmochine + quinine	80	NR
Gribben 1933, Trinidad and Tobago [28]	NR (259 cases treated during October–December 1931; and 96 cases treated during July 1932	Before and after study (compared malaria cases seeking treatment before MDA and after MDA)	Plasmoquine + quinine	80	Lake draining and oiling
Henderson 1934, Sudan [29]	NR. Heavily infected village	Non-RCT. 160 members of an isolated community in Sudan (~1/2 of population)	Quinoplasmine + plasmoquine	100	Larviciding
White 1934, India [60]	55%	Non-RCT Children of age 0–10 years living in a railway settlement and an adjacent village were selected for both control and intervention	Euquinine, plasmochin	94	Larviciding
Ray 1948, India [61]	0.7% in adults to 35% in children	Before and after study conducted in tea estate in India	Paludrine	75	IRS
Banerjea 1949, India [62]	4.37%	Non-RCT. The study was conducted in rural west Bengal	Proguanil	89	NR
Van Goor 1950(C), Indonesia [63]	26 5–40%	Before and After study Different study arms examining the efficacy of monotherapy mainly with varying prophylactic doses of proguanil (a small subgroup was given chloroquine). The goal was to establish an optimal dosing regimen	Proguanil and chloroquine	90	NR
Norman 1952, India [64]	NR	Descriptive The chemoprophylaxis in Assam Valley tea estate populations (laborers and their families) was conducted for 6 months. Half of the populations did not take the medicine as evidenced in their examined urine	Proguanil	52	NR
Archibald 1956, Nigeria [65]	48.7% (5–10 years)	Non-RCT Children with 5–10 years age group were selected for the chemotherapy	Pyrimethamine	80	NR
Clyde 1958, Tanzania [66]	82.6%	Before and after Labourers on tea estate in eastern Usumbara Mountains	Pyrimethamine	82	IRS
Van Dijk 1958, Netherlands New Guinea (PNG) [67]	11.2%	Before and after Inhabitants of 30 villages in Demta district	Chloroquine	93	IRS with DDT
Afridi 1959, Pakistan [68]	NR	Non-RCT Population at villages near Daur river in Hazara district of west Pakistan	Pyrimethamine	96	NR
Van Dijk 1961, Netherlands New Guinea (PNG) [69]	17.5%	Before and after Inhabitants of Inanwatan. Area is unstable hyperendemic	Chloroquine	97.2	NR
Metselaar 1961, Netherlands New Guinea [70]	12–28%	Before and after The population lived near lake Sentani and were likely to sleep in garden houses	Chloroquine + pyrimethamine	90	IRS with DDT
Ho 1965, China [71]	NR	Descriptive Mass screen and treat study	Chloroquine + primaquine, pyrimethamine + primaquine	95	ND
Ossi 1967, Iraq [72]	NR Annual incidence of malaria cases 2221	Before and after Chemoprophylaxis was conducted in Basrah city with the highest malaria incidence	Chloroquine + pyrimethamine	57 5	IRS with DDT
Singh 1968, India [73]	0.98 cases/1000/population/month	Before and after All people in 29 villages in Azamgarh but in subsequent rounds only targeted febrile cases, their contacts and housemates	Chloroquine, primaquine	72.7	IRS with DDT
Lakshmanacharyulu 1968, India [74]	56.3%	Before and after Labourers within a dam and canal project area previously not know to be malaria endemic	Chloroquine + pyrimethamine	80	IRS with DDT, larviciding
Onori 1972, Syria [75]	Average monthly number of malaria cases: 46.6	Before and After All individuals in Ghab Village	Chloroquine + pyrimethamine	85	IRS with DDT
Najera 1973, Nigeria [76]	19%	Non-RCT All villagers older than 3 months from Kankiya district in North Central State	Chloroquine + pyrimethamine	85.9	IRS with DDT
Schliessmann 1973, Haiti [77]	0.02/1000 population/month	Descriptive No details on drug or the regimen Population of 3 localities within Section Rurale I Varreux	NR	40	IRS with DDT
Paik 1974a, British Soloman Islands [78]	27.8%	Before and after study Only children aged 2–9 years of north coastal areas of Nggela island group	Chloroquine + pyrimethamine	90	IRS with DDT
Paik 1974b, British Soloman Islands [78]	18/1000 population/month	Before and after Study Population of Gilbertese settlements in western district. Sleeping outdoor is common here	Chloroquine + primaquine	90	NR
Kondrashin 1985, India [79]	In 1980: Pv: 3.4/1000pop/month and Pf: 1.9/1000pop/month	Before and after Inhabitants of 8 PHC (Primary Health Centre) catchment area in five malaria endemic districts of Andhra Pradesh	Chloroquine + primaquine	85	IRS
Strickland 1986, Pakistan [80]	24.9%	Before and after All individuals above age 3 years. Suspected cases of malaria were treated with chloroquine and individuals with parasitemia were treated with Sulfadoxine and Pyrimethamine	Sulfadoxine, pyrimethamine	67.3	NR
Hii 1987, Malaysia [81]	46.3–55.6%	Before and after The intervention intended to determine the effects of impregnated bed nets. Improver and lack of use and damage to the bed nets and reluctance of the population to take the drugs were noticed	SP + primaquine	81.6	ITNs
Babione 1996, Central America (multiple locations) [82]	NR	Descriptive Field test in El Salvador. Population and intervention not well described	Chloroquine + primaquine	77.5	IRS with DDT, larviciding

**Table 3:** Studies documenting community engagement activities only (n = 12, in chronological order)

Author, year and country	Epidemiology (baseline parasitemia)	Study type	Anti-malarial	Additional interventions
Butler 1943, South Pacific [36]	12–16%	Before and after Study was largely a review of the Us Navy’s malaria control program implemented on an unspecified occupied island in the South Pacific. Natives of 2 villages were recruited into the study	Mepacrine	NR
Berberian 1948a, Lebanon [26]	NR	Non-RCT All persons aged above 6 months of Saideh village	Chloroquine	NR
Chaudhuri 1950a, India [83]	50%	Non-RCT Only children were participants in the study	Proguanil	NR
Edeson 1957, Malaysia [84]	29%	Non-RCT All ages except infants. Inhabitants of rural rice growing area of Negri Sembilan state	Proguanil	NR
Gabaldon 1959, Venezuela [39]	0.24%	Before and after Population of eastern and western Venezuela. All persons aged >1 month	Pyrimethamine	IRS with DDT
Clyde 1961a, Tanzania [32]	Morbidity rate: 76.1% Parasitemia in european children: 11.5%	Before and after Europeans living in Tanga	Quinine	Bed nets
Charles 1962, Ghana [33]	55.3%	Before and after All aged residents of Akrofu villages	Pyrimethamine	NR
Sehgal 1968, India [37]	1.8%	Before and after All individuals except the very sick, old and the pregnant women	NR	Irs with ddt
Omer 1978, Sudan [85]	40.5%	Non-RCT All residents of 4 villages in Gezira ecological area of Northern Sudan	Chloroquine	NR
Maccormack 1983, Tanzania [47]	NR	Non-RCT The age range of the participants was 0–15 years	Chloroquine	NR
Dapeng 1996, China [38]	0.8/1000 population/month in 1984	Before and after All residents within high incidence areas in 4 counties in Xinyang prefecture	Chloroquine + primaquine	Irs with ddt, itns
Song 2010, Cambodia [43]	2–50%	Before and after All residents above 1 year from 17 villages with parasite rates 20% or higher in Kampong Speu Province	Artemisinin + piperaquine + primaquine	NR

**Table 4:** Studies documenting both community engagement and population coverage (n = 11, in chronological order)

Author, year and country	Epidemiology (baseline parasitemia)	Population coverage/%	Type of study and the context	Anti-malarial(s)	Additional interventions
Archibald 1960, Nigeria [45]	48.7%	89.7	Non-RCT Inhabitants of villages in Western Sokoto malaria control campaign	Chloroquine + pyrimethamine	
Clyde 1962, Tanzania [46]	59.8–64%	95	Before and after Participants of all ages were recruited	Amodiaquine + primaquine	IRS with DDT
Roberts 1964a, Kenya [40]	23%	95	Non-RCT Participants of all ages were recruited and were from highlands in Kenya from 2 districts	Pyrimethamine	
Garfield 1983, Nicaragua [27]	0.42–0.83 cases/1000 population/month	70	Before and after (nationwide) Medicine was administered to all persons aged >1 year over 3 days given once to the entire population	Chloroquine + primaquine	
Baukapur 1984, India [34]	0.18/1000 population/month	76	Before and after All persons in 2 villages in Valsad District in Gujarat. Migratory population came from malarious areas during study period	Chloroquine	NR
Pribadi 1986, Indonesia [31]	13.2%	93.7	Before and after Mass chemoprophylaxis among all the individuals in Berakit village	Chloroquine	
Doi 1989b, Indonesia [44]	30%	100	Before and after All aged participants were treated	SP + primaquine	NR
Kaneko 2000, Vanuatu [35]	62%	88.3	Before and after MDA was conducted with all individuals in Aneityum islands	Chloroquine + primaquine + SP	
Von Seidlein 2003, Gambia [42]	41.6%	85	RCT/Cluster RCT Individuals older than 6 months and all non-pregnant women in 9 villages and placebo in other 9 villages	AS + SP	
Shekalaghe 2011, Tanzania [12]	0% by microscope and 2.6% by PCR	93	RCT/Cluster RCT Individuals above 1 year were included. People who had received ACTs in 2 weeks prior to the study, pregnant women and people with anaemia received different drug regimen. Four each intervention and control randomly assigned	AS + SP + primaquine	Vector control, larviciding
Lwin 2015, Thai–Myanmar border [25]	7.3% by microscopy and 18.4% by uPCR in one study village	40	Before and after Study population consisted of all the villagers excluding children <14 years and pregnant women in Thai–Myanmar border	Dihydro-artemesinin + peperaquine	NR

### Community engagement data extraction and analysis

Of the 103 primary articles, 47 were excluded after a preliminary review because they did not describe population coverage or community engagement activities (Additional file 2). Among the remaining 56 articles, six described community engagement only very briefly and were excluded [24]. One additional article was added during the update of the review [25] resulting in 51 articles retained for analysis. A thematic analysis was conducted on a total of 23 articles, which described community engagement activities. These included 12 articles that described community engagement activities only and 11 articles that described population coverage and community engagement activities.

## Results

### The nature of the reviewed articles

Fifty-one articles reporting the results of studies conducted in 26 countries were retained in the analysis. Population coverage without community engagement was documented in 28/51 (55%) papers (Table 2). Twelve of 51 (24%) articles described community engagement activities in detail but not population coverage (Tables 3, 5), whereas 11/51 (22%) articles described community engagement activities in detail and population coverage (Tables 4, 6). Among all articles reviewed (n = 51), median quartile of the studies were conducted before 1965 (Q1 = 1956, Q2 = 1965 and Q3 = 1984).

**Table 5:** Studies documenting community engagement activities only (n = 12, in chronological order)

Author, year, country	Community engagement				Other relevant factors
	Health education	Incentives	Community (health) structures	Human resource mobilization	
Butler 1943, South Pacific [36]	Bulletins, announcements, short talks and movies	NR	The medical officer provided direction and information dissemination	Local community members provided manual labor	Mild initial intolerance to the anti-malarial consisted mainly of nausea, vomiting and diarrhoea. Less than 1% of participants showed absolute intolerance, usually manifested by repeated vomiting
Berberian 1948a, Lebanon [26]	NR	NR	Discussion with village head and elders were held and the study was started after their consensus	NR	Villagers were grateful and demanded for the anti-malarials to an extent that villagers in the control arm were also provided with the anti-malarials which reduced the people in control arm. The population was mobile. For instance, only 160 out of 200 were present in one of the village
Chaudhuri 1950a, India [83]	NR	NR	Young men from the village established themselves to form an anti-malarial society and were affiliated to central anti-malarial society	A local man worked as a volunteer to visit door to door of the villagers. A filed assistant was appointed for drug distribution	Some villagers migrated out of the village because of the perennial fear of malaria. Within the village, some villagers were reluctant to swallow the tablets in front of the study staff and preferred to keep it to take later. Adverse events such as vomiting affected others from taking them. There were propaganda about the ill-effects of the medicine which was eventually resolved
Edeson 1957, Malaysia [84]	NR	NR	In each valley, committees were formed to serve as channels through which villagers were informed about the blood surveys or house spraying and villagers could express their views to the committee as well	Village volunteer was responsible for drug distribution	Even though medicine were distributed by a volunteer, there was no actual supervision of participants taking antimalarial)
Gabaldon 1959, Venezuela [39]	NR	Incentives (lottery tickets) were provided for those completing MDA. A bonus incentive was given to MDA distributors if their sector was found malaria free	Nurses at local dispensaries coordinated with the study in keeping the record of any cases of malaria and preparing the slides	Involvement of rural visitors as staff (two types: drug dispensers and blood slide makers/collectors) who were supervised by inspectors and sub-inspectors A local doctor was engaged in conversation with few villagers who were reluctant to take the medicine	There were relapses observed in the groups of 5–14 years and 15 years or more after the completion of MDA. These groups took less than designated 18 treatments. This was attributed to the greater mobility, consequently, it was difficult to find them at their houses
Clyde 1961a, Tanzania [32]	Articles for general public were written in 2 local newspapers	NR	German Hospitals as health structures were already present	The medical facilities and treatment was initiated by German health workers and in the established hospitals by Germans	Government’s consensus was sought for the initiation of Malaria control program in Tanga (research site)
Charles 1962, Ghana [33]	Weekly health education class was conducted. Residents were also prepared by preliminary educational propaganda	NR	The trial formed part of the pilot malaria eradication project supported by Ghana government, WHO and UNICEF	Anti-malarial distribution was delegated to the formed team consisting of 2 volunteers who were selected from representative clans of the community	The study town had successfully participated in previous community development projects and the community was deemed to be cooperative. However, reluctance to take the medicine was noticed particularly in children and some informed that the tablets were sold or shared with other villagers
Sehgal 1968, India [37]	Health education through audio-visual aids and using local literature (language). Intimate and personal persuasion was applied for resisting tribal population	Incentives were paid as an advance for building houses	Central to community level health structures and social structures were utilized which included additional staffs recruitment at various positions	Augmentation of staffs were done in existing positions. In addition, lower qualified community members were recruited for the work	Geographical inaccessibility was a major barrier for the malaria control program. Reluctance of staffs to work and reluctance of tribal population to the intervention were major barriers which were subsequently resolved
Omer 1978, Sudan [85]	NR	NR	Ministry of Health was involved in providing the technicians for the check-up of blood slides. Local health structure such as public health office, local school and youth organizations were directly involved	A school teacher was asked to supervise who was under resident public health officer supervising the operation. 2 or 3 people, generally from the youth organization, helped the night before and in the morning to encourage people to participate	The people in the village were cooperative and appreciated the medical services rendered to them during the previous survey. The purpose of the chemoprophylaxis was explained to them (but no details on how was it conducted) and they volunteered to cooperate
MacCormack 1983, Tanzania [47]	Health education through meetings was delivered in tier approach to key community persons who in turn educated families	NR	Anti-malarial supplied by WHO, government committed to the Malaria control plan and health structures present at the rural site coordinated with the project	The direction and operation of the project was taken care by the medical director of the hospital in the study area. Officials and staffs at local hospital were involved	As many as 28% of children complained of vomiting and 56% complained of itching, and other unfavourable qualities of chloroquines were indicated for the reluctance to adhere to the medicine
Dapeng 1996, China [38]	Before introducing the malaria control program in the community, health education through the primary health care system, by means of meetings, films, posters, and videos were conducted thus encouraging villagers to participate	NR	Malaria control program was carried out through the existing primary health care system already in place	Additional experts from the provincial and central level were involved in field research, guidance and evaluation. Village doctors were responsible for the chemoprophylaxis and the clinical care of the patients	The control program involving malaria treatment and chemoprophylaxis was less successful than the vector control. Bed net impregnation was more accepted in the community than DDT spraying as it killed flea, lice and bedbugs as well
Song 2010, Cambodia [43]	Village leaders cooperated in educating the general (study) population	NR	Local health workers from the community and volunteers from the village were involved in the study	Village malaria workers were recruited to distribute drugs and monitor drug administration	The anti-malarial were redistributed in 4 of the study villages because of the lack of anticipated reduction in malaria. Improper distribution and inadequate training of VMWs were attributed for the lack of reduction and some VMWs were subsequently replaced

*NR* not reported, *IRS* indoor residual insecticide spraying, *ITN* insecticide treated bednet, *DDT* dichloro diphenyl tricholoroethane, *ND* not done

**Table 6:** Studies documenting both community engagement and population coverage (n = 11, in chronological order)

Author, year, country	Health education	Incentives	Community (health) structures	Human resource mobilization	Other relevant factors
Archibald 1960, Nigeria [45]	NR	NR	Cooperation was sought with the village authorities (local native authority and the Emir in Council of 3 study village). The purpose of the study was explained to local councils and meetings were conducted with the family heads	The drug administration was carried out by authorities in-charge and staff (medical officer, the superintendent of rural health and the health sister) at the local health care centre The local drug distribution team consisted a team leader, a female community attendant, a native authority representative from each ward and the locally recruited labourer	The acceptance of the small tasteless tablet of pyrimethamine was far better than the chloroquine which was often vomited by toddlers. The bitter taste, big size and the number of chloroquine tablets all were disadvantage for the administration
Clyde 1962, Tanzania [46]	Information dissemination through health education sessions and demonstration of the advantage of the project	NR	Cooperation of the public was achieved through involvement of community leaders. Community leaders were sought for any raised problems and various reasons for defaulters	A medical worker paid visits for treatment on a personal basis to everyone in the study site. Persistent follow up of defaulters was carried out	Authors attributed the success to correct approach to the villagers through the community leaders and promotion of awareness through health education and demonstration of the advantages of the project
Roberts 1964a, Kenya [40]	NR	NR	A joint collaboration with Ministry of Health of Kenya, WHO and UNICEF. Malaria control was handed over to the district health office (medical officer). Propaganda was introduced about the study, meetings were held with the inhabitants such as at trading centers, dispensaries and schools. A concerted program was organized to inform all the population about the objective of the campaign	The advisory capacity was provided from the medical headquarters and ministry of health. The daily operation of the project was carried out by the district health inspector School masters and agricultural scouts were involved as monitors and guides. 100 men were selected, trained and recruited on the work	A joint collaboration and successfully devolved the responsibilities to host government and local structures
Garfield, 1983, Nicaragua [27]	Literacy follow up classes, health promotion activities were conjoint work with other diseases too	NR	Malaria control program in Nicaragua was one of a series of national health campaign	70,000 anti-malaria volunteers trained to conduct a census, provide door to door education about malaria, promote community participation, package and distribute drugs and keep records. In addition, about 10% of the country’s total population was estimated to have taken part in promotional activities	Apart from the coverage of 70% of the whole population, the other benefits were the value of national level census, the long term impact of modifications in malaria control strategies based on campaign results, and the impact of increased citizen awareness on case finding and malaria control efforts
Baukapur 1984, India [34]	Health education was carried out at different levels. Community health volunteers played a major role in increasing the awareness in the community	NR	The local health structure and additional health personnel and resources from district headquarters were utilized in this program	Trained microscopists, leave reserve staffs, community health volunteers, malaria staffs from the district headquarter to local structures were involved in the malaria control program	The study utilized both local and district level health staffs in both technical and non-technical works (health education) related to the malaria control work
Pribadi 1986, Indonesia [31]	A comprehensible learning module for the community was prepared to provide the health education. Health education to children were also provided by the village teachers (cadres)	NR	The regular monthly meetings of the cadres and the periodical meetings with the health centre officer was conducted concerning the cases of malaria. The health centre and the sub-health centre both were provided with a paramedic who was trained at blood slide preparation and treatment	Nine key persons from the sub village, consisting of school-teachers, heads of the RT’s and active young people were selected (cadres/facilitators) who were chosen by villagers co-ordinated by village heads. The cadres were trained on malaria signs and symptoms and treatment. The volunteers/cadres were appointed to distribute the learning module to each house in the village	Adverse events were reported from some villagers and discussions were held to counteract the misunderstandings and were treated at local health center
Doi 1989b, Indonesia [44]	NR	NR	Malaria control was one of the activities among other community health activities. A local school teacher, health center staffs and village volunteers were involved in the implementation of the project	Part of a national initiative. A health center staff was responsible for testing the blood samples, examination of spleen and similarly, two community health volunteers were responsible for recorders and guides	The project operated under the national initiative and the size of the project within the national initiative was proportionately small
Kaneko 2000, Vanuatu [35]	Aggressive health education were conducted and were attributed for the sustained compliance with the bed net programme. Information dissemination on MDA medicine through meetings were conducted after the incidence of adverse events	NR	The district malaria supervisor and the staff of the central malaria section directed the local malaria intervention activities. Several meetings with community were conducted to explain the purpose and the objective of the MDA.	12 village volunteers were selected and trained as MDA staff and were responsible for drug administration. One village volunteer was selected by the local health committee for the training of malaria microscopy. Thus trained community microscopist and registered nurse cooperated to guard against the future introduction of malaria	The adverse event such as vomiting and the number of tablets decreased compliance. The meeting was held to answer their questions and additional information was provided to the villagers. In addition, Chloroquine was removed from the regimen after 4^th^ round
Von Seidlein 2003, Gambia [42]	NR	NR	Discussion and co-operation with national and district level, members of the government district health team was sought Meetings were conducted in villages in the presence of national and district level government officials to explain the objective and methods of study and in addition, questions were answered during the meetings Village elders were engaged in the meetings to discuss about participation at the MDA. The consent of participation was given by the village head	A translator and fieldworker was appointed for consent process with the participants during drug administration. Similarly, two health center staff recorded the visits of individuals living in the study village	Because of likely human and mosquito movement between villages, apart from 9 each intervention and placebo-controlled villages, 24 other neighbouring villages were treated which were not included in the study
Shekalaghe 2011, Tanzania [12]	NR	NR	Meetings were conducted in all villages and the study was explained to all village leaders and local ten-cell leaders (*balozi)*. A stepwise consent process was applied. At first, the verbal approval was obtained from these local leaders. Verbal household consent was sought after explaining about the study at their household. Three health facilities in the study area were involved in passive case detection The concept of asymptomatic malaria and the benefits of participation was discussed during community meetings	Seven study teams consisting of one medically trained individual (medical doctor or medical officer) and 1 or 2 field workers were responsible for consenting, screening participants for the safety and administering drugs All *balozis* were involved in the monitoring of movement of people in the study area. The approach to give local leaders a central role in explaining the study purposes, drug administration and monitoring migration were considered to influence the community participation	Each household received a household ID card and were entitled to free health care at the local health centers Number of tablets (24 tablets/3 days; 7/day for 2 days and 10 for 3rd day) were complained as burdensome by some participants A symptom diary was provided to the parents for their children, where they were asked to record the daily occurrence of fever, headache and body pains
Lwin 2015, Thai-Myanmar border [25]	NR	NR	The cross-sectional survey results were discussed with the communities and different containment strategies were discussed. Chemoprophylaxis was endorsed by the residents of the village. Community leaders and key workers were consulted about the project, and approval was obtained from the Tak Province Community Ethics Advisory Board (T-CAB). The T-CAB consists of representatives from both the local ethnic Karen and Burman border communities which has been the primary ethics review body for health care interventions along the border for past 6 years	Village malaria workers were trained in detection and treatment	Community participation was low mainly because of difficult access to the communities, which resulted from the terrain and political instability

*NR* not reported, *IRS* indoor residual insecticide spraying, *ITN* insecticide treated bednet, *DDT* dichloro diphenyl tricholoroethane, *ND* not done

The studies were conducted in contexts with diverse healthcare infrastructure, health needs, population mobility and population size. For instance, the study in Lebanon involved study villages, which were deprived of healthcare centres (physicians or pharmacies were unavailable within 18 km of the village). In addition, the study population was mobile as they often leave the village in search of job and to escape the winter [26]. This had implications in terms of villagers’ willingness to follow study instructions, such as not to take other anti-malarials apart from the study drug, chloroquine.

In some cases, mass anti-malarial administration was conducted as part of research in selected villages, whereas, in others, MDA was part of a national health campaign. An example of the latter is Nicaragua where the entire population was treated. High level of community participation in a literacy campaign in previous year was a stimulus for the nationwide MDA. Prior to mass anti-malarial administration, health campaigns had been conducted for polio vaccination, sanitation and environmental hygiene, rabies control in domestic pets and vaccination against diphtheria, pertussis and tetanus [27].

### Population coverage

The 28 studies that documented only population coverage were published from 1931 to 1996 (Table 2). The mean reported population coverage in these 28 studies was 82% (range 40–100%), with seven (25%) studies reporting a population coverage below 79% (Table 2). The 11 articles reporting both population coverage and community engagement activities were published from 1960 to 2015 and described, on average, 84% coverage (range 40–100%). Three of the 11 (27.3%) studies reported a population coverage below 79% (Table 4). It was often unclear or not specified how the coverage was estimated. For example, in Trinidad and Tobago, 2142 people were given plasmoquine and quinine, but only 1289 people completed the full course. In this study, the coverage was not reported and the selection of the target population was not mentioned [28]. Similarly, in Sudan, the anti-malarials were reportedly taken by all members of the targeted population but no details of coverage, population size or the selection procedure were provided [29].

### Community engagement activities

This section describes the type and process of community engagement activities: health education, provision of incentives, utilization of existing community structures, human resource mobilization and steps in community engagement.

#### Health education

Of the 23 articles (Tables 5, 6) with detailed descriptions of community engagement, 12 focused on health education employing the following media and methods: (1) newspapers, written leaflets and posters, (2) audio-visual materials, such as news-bulletins, movies and videos, and (3) meetings with key community persons, announcements in health facilities in conjunction with community members and involvement of local health workers, government health officials and village volunteers.

Two articles, reporting on studies in Nicaragua [30] and Indonesia [31] incorporated large-scale health education on malaria and MDA. Health education was conducted in various different contexts and various methods were applied. For instance, in Tanzania, articles for the general public were written in two local newspapers [32]. In Ghana, weekly health education classes were conducted among the residents and they were asked to spread the message to other villagers [33]. In India, health education sessions were conducted in different levels where community health volunteers played a major role in increasing the awareness in the community [34]. In Vanuatu, intensive health education classes were conducted after coverage dropped and in subsequent round coverage improved [14, 35]. Information dissemination was conducted through newspapers [32], pamphlets and news bulletins [36]. In two studies [37, 38] audio-visual materials were employed to explain the planned intervention [37]. In China, the health education was delivered through the primary health care system by the means of meetings, films, posters and videos [38].

#### Incentives

Two articles documented the use of incentives in MDA but they did not report the corresponding population coverage [37, 39]. In Venezuela, MDA participants received incentives ranging from a lottery ticket to sugar candy on completion of the entire anti-malarial course. In addition, financial incentives were provided to MDA staff for each malaria positive slide [39]. In India, financial incentives—an advance for housing construction—were given to reluctant tribal populations [37].

#### Community structures

Community structures consisted of existing organizations, physical infra-structure, health care providers and health service centres. The involvement of community structures was often a core component of introducing the MDA to the target communities. In India, personnel from the local health centre and additional staff from the district assisted in conducting the MDA [34]. In Kenya, the MDA was implemented in the community through the local district health office [40]. Apart from conducting community meetings and utilizing community structures, a study [25] in Thai–Myanmar border areas utilized an existing community ethics advisory board (Tak Province Community Ethics Advisory Board [T-CAB]) [41].

#### Human resource mobilization

Human resource mobilization varied from the simple involvement of community members or health staff to assist with the study to complete hand over of the MDA operations to government staff. For instance, in the Gambia, a translator and a fieldworker were appointed for consent process with participants whereas the staff at health centre recorded the visits of villagers at health centre [42]. In Cambodia, village malaria workers were recruited to distribute the drugs and monitor drug administration [43]. By contrast, in Nicaragua, a nationwide malaria control program recruited 70,000 people as anti-malaria volunteers who were trained to conduct the census, provide door to door education, promote community participation, distribute drugs and keep records [27]. On Aneityum Island, Vanuatu, village volunteers were selected and trained as MDA staff and were responsible for drug distribution [35].

#### Steps in community engagement

Community engagement was often a process and the MDAs involved participants in various ways throughout the duration of the intervention. For instance, in The Gambia, researchers first discussed the project with and sought cooperation from national and district-level members of governmental and health structures. Subsequently, meetings were conducted in the target communities with village elders and other prominent residents in the presence of the government officials [42]. With the consent of village leaders, meetings were held with community members to introduce the purpose and details of the study, which was then followed by the individual consent process. On Aneityum Island, the MDA plan was first formulated by the malaria control programme at the Department of Health. Operationally, MDA was led by the district malaria supervisor and several meetings were held with the communities to explain the MDA’s purpose and procedures, and to elicit the community’s full commitment to eliminate malaria from the island. Subsequently, 12 village volunteers were selected and trained as MDA staff and anti-malarial administration was carried out with the supervision of a registered nurse who was responsible for healthcare on the island, the district malaria supervisor and central malaria section staff [14, 35].

### The relationship between community engagement and population coverage

Among the 11 studies (Tables 4, 6) that documented both community engagement activities and coverage, coverage ranged from 40 to 100% [25, 27, 34, 35, 40, 42, 44, 45]. The MDA with the highest reported population coverage (100%) was part of the North Sumatra Health Promotion Project, Indonesia and was on-going for 10 years. In this national initiative, existing village health centres and staff were responsible for the activities, such as collecting fingertip blood samples and examining spleen size. Two village volunteers also worked as recorders and guides and the primary school teacher assisted in identifying and examining pupils [44].

Another successful MDA that reached 95% of the target population was conducted in Kenya, as part of chemotherapeutic campaigns in 1953–1954 and as a joint collaboration between WHO, UNICEF and Ministry of Health. In this case, the district health offices were responsible for overall organization and supervision of the MDA and the district health inspector was charged with the daily running of the operation. Most community leaders, such as administrative chiefs, headmen, school teachers and clerks were involved [40].

On Aneityum Island, mass anti-malarial administration contributed to the elimination of malaria from the island. Here, the department of health was closely involved: the district malaria supervisor took charge of the programme and delegated responsibilities to local staff from government health facilities. Twelve community members were recruited and trained as MDA staff who carried out day-to-day activities, such as drug distribution, recording and supervision. The MDA compliance rate decreased from 90 to 79% in the 2nd and 3rd rounds; this resulted from the adverse events related to chloroquine and the number of tablets participants had to take and, in response, community meetings were held. Additional information was provided and chloroquine was removed from the scheduled anti-malarial regimen in the 5th and 9th rounds after discussion with the community [14, 35].

Amongst the articles that reported coverage and community engagement activities, the lowest coverage −40%-was recorded on the Thai–Myanmar border [25]. Initially, the mean coverage in three villages for the malaria parasite survey was 77%. After discussing the results with community leaders and the T-CAB, MDA was agreed as an intervention. Two of the three villages were inaccessible during the rainy season and so anti-malarials could not be administered there. The low coverage was explained in terms of political fragmentation, inaccessibility and high mobility [25].

## Discussion

This review has identified and examined reports of mass anti-malarial administrations that described community engagement activities and/or population coverage. Conducted between December 30, 1931 and August 16, 2015, MDAs varied widely by anti-malarial regimen, study type, context, community engagement activities, and population coverage. In the following sections, the research questions are discussed with particular regard to the limitations of the reviewed articles.

### Population coverage

Amongst the reviewed articles, the mean population coverage for the mass anti-malarial administrations was above 80%, the suggested minimum to interrupt malaria transmission [6]. Amongst the articles that described population coverage and community engagement activities, the mean was even higher. Furthermore, when community engagement involved government and local community structures, coverage was above 85% (range = 70–100%) [27, 34, 35, 40, 42, 44, 45] and when community engagement activities depended on the community alone, mean coverage was over 80% (range = 40–95%) [12, 25, 31, 46]. These figures suggest cause for optimism with regard to reaching the levels of coverage required to eliminate falciparum malaria from the Greater Mekong sub-region.

The reporting of how population coverage was calculated was, often absent or inadequate; little detail was offered on the denominator or numerator populations, who was excluded, for example, from anti-malarial administration, or how the total target population was calculated, with regard to including or excluding mobile groups. The absence of such detail limits direct comparisons across the studies. Ideally, future research on mass anti-malarial administration should report its fullness, the methods used to calculate population coverage.

The reviewed articles also provide few references to participants’ adherence to the anti-malarial regimen [31, 35, 36, 45, 47]. Adherence is likely to be a particular challenge when administering a multi-dose multi-day anti-malarial regimens. Although adherence can be ensured by delivering anti-malarials as DOT, this is likely to present challenges when part of large scale MDA programme. Low-prevalence settings, such as those in the Greater Mekong sub-Region, are also likely to present potential challenges for adherence if DOT is not possible. Future research should address the question of MDA adherence in areas of low clinical malaria prevalence.

### Community engagement activities

The reviewed articles described community engagement activities that included the simple provision of information about the MDA to the community, the utilization of existing community structures for MDA implementation, delivery of health education, and building local human resource capacity through training. Some articles provided detailed accounts of the community engagement [14, 30], overall, there was a general lack of detail regarding the approach to community engagement and the activities conducted.

Community engagement approaches have been categorized as top-down (vertical) and bottom-up (horizontal) [20]. Many of the reviewed articles reported a combination of these approaches [27, 34, 35, 40, 42, 44, 45] and community engagement was often a process, involving multiple steps. First, the top-down approach was used to garner support of officials and gain access to the research site [48, 49]. In light of the criticisms of such an approach and the need to gain the consensus of the community, more bottom-up activities were subsequently utilized [50–52]. This is particularly the case in more recent MDAs [48, 53–55]. In many of the reviewed articles, [12, 27, 31, 34, 35, 40, 42, 44–46], the use of existing community structures, the local health system and involvement of the community in meetings, health education and training of the local staff were essential features.

Bottom-up approaches to community engagement include community-directed interventions (CDI), which entails community members taking an active role in the planning and execution of an intervention. For MDAs, such activities include carrying out the target population census, distributing drugs, mobilizing other community members and recording treatments provided [14, 27, 43]. In the reviewed Vanuatu study, 12 village volunteers were selected and trained as MDA staff who delivered each MDA dose under supervision. A multi-country review of CDIs to control and manage tropical diseases, including malaria, identified this approach as more effective at increasing coverage and achieving the targeted outcome compared to conventional vertical approaches [21, 22]. This approach was also more cost effective and operationally efficient with community members’ enthusiasm described as pivotal in increasing feasibility and sustainability [22].

Analysing and comparing the community engagement activities is complicated by the diverse study conditions under which the MDAs were undertaken. These included controlled trials, which entailed close monitoring of participants, whereas others were less intensive implementation studies. Aspects of these controlled clinical trials, such as the provision of incentives (and/or healthcare), would not be feasible as part of a larger implementation and only qualified lessons can be drawn from such studies. With regard to incentives, during a multi-country study in Africa, the effects of different incentives types were compared: material incentives, such as gift items, money or T-shirts were less effective than “intrinsic” incentives, such as sense of recognition, the feeling of making a worthwhile contribution and knowledge gained [21, 22].

In most of the reviewed MDAs, community engagement activities focused on promoting the population coverage and facilitating study operations. However, for many researchers, community engagement now encompasses the broader aim of promoting ethical global health research through, for example, socially responsible knowledge production, capacity development and health promotion, such as through changes in health behaviour [19, 56, 57]. From this perspective, community engagement cannot be simply evaluated in terms of its contribution to the study aims, in the case of mass anti-malarial administration, mainly population coverage.

The local social and cultural context also influences MDA coverage and adherence in various ways: for example, community members’ familiarity with anti-malarials, perceptions about the seriousness of malaria and its local prevalence can influences people’s readiness to take medicines when apparently healthy. The nature of the anti-malarial, particularly any adverse events that are associated with it and how they are interpreted with regard to local understandings of illness, is also likely to influence community responses to the intervention. Similarly, the local availability of healthcare prior to the study could influence participation. Political divisions within the target community, perceived or enacted nepotism can foster resentment and objections towards MDA.

### Strengths and limitations

This review is the first comprehensive analysis of the published literature on community engagement and population coverage in mass anti-malarial administrations. The findings are limited by the relatively low proportion of articles that documented community engagement activities (45%; 23/51). The findings are also limited by the heterogeneity of study methods, population and study sites. This is particularly relevant in terms of the methods used to calculate population coverage, which was often reported with insufficient detail to ensure fair comparisons.

## Conclusion

The reviewed MDAs varied widely by anti-malarial regimen, study design, context, era, community engagement activities, and population coverage. The mean population coverage for the reviewed mass anti-malarial administrations was above 80%, but inadequate reporting of coverage calculation methods complicates comparisons between studies. Various community engagement activities and approaches were described, many of which contained limited or no details. Community engagement plays a major role in achieving high population coverage in mass anti-malarial administrations. Amongst the reviewed articles, coverage was highest when community engagement involved government and community structures, such as in Nicaragua and on Aneityum island. Other factors, such as the social, cultural and study context, may also play an important role but were not investigated by these articles. More research is needed to disentangle the factors influencing population coverage, adherence, “success” in mass anti-malarial administrations and the role of community engagement activities and approaches. Future research must also (1) report in full the methods used to calculate population coverage; (2) provide details of community engagement activities; and (3) evaluate the impact of the local social context as well as the particular community engagement activities and strategies.

## Additional files

**Additional file 1.** Literature search method.

**Additional file 2.** Studies that did not document either community engagement or population coverage (n = 47).
