# Supplementary material for: Community engagement and population coverage in mass anti-malarial administrations: a systematic literature review
Source: Malar J. 2016 Nov 2;15:523. doi: 10.1186/s12936-016-1593-y (PMC5093999; doi:10.1186/s12936-016-1593-y)
Supplement: Supplementary file 1 — Additional file 1. Literature search method. [file 12936_2016_1593_MOESM1_ESM.docx]

**Additional file 1: Literature search method**

Search method (as originated from Cochrane review)

Databases searched: Cochrane Infectious Disease, Group Specialized Register; Cochrane Central Register of Controlled, Trials (CENTRAL), published in The Cochrane Library; MEDLINE+; EMBASE; CABS Abstracts; and LILACS.

The final search was conducted on 23^rd^ June 2015.

Search strategy:

MEDLINE+

A. Anti-Malarials

exp Antimalarials/ or exp Malaria/ or antimalarial* or anti-malarial* or ((schizonticidal* or gametocidal* or hypnozoiticidal* or drug*

or treatment) and (malaria*))

B. Mass Administration

((mass or coordinate*) adj5 (administ* or distribut* or applicat* or “use” or therap* or treatment*))

EMBASE

A. Anti-Malarials

exp antimalarial agent/ or exp malaria/ or antimalarial* or anti-malarial* or ((schizonticidal* or gametocidal* or hypnozoiticidal* or

drug* or treatment) and (malaria*))

B. Mass Administration

((mass or coordinate*) adj5 (administ* or distribut* or applicat* or “use” or therap* or treatment*))

COCHRANE LIBRARY

A. Anti-Malarials

(Must run each MeSH term separately. Ovid syntax used for recording purposes.)

exp Antimalarials/ or exp Malaria/ or antimalarial* or anti-malarial* or ((schizonticidal* or gametocidal* or hypnozoiticidal* or drug*

or treatment) and (malaria*))

B. Mass Administration

((mass or coordinate*) near/5 (administ* or distribut* or applicat* or “use” or therap* or treatment*))

CAB DIRECT

A. Anti-Malarials

ti=(antimalarial* or anti-malarial* or ((schizonticidal* or gametocidal* or hypnozoiticidal* or drug* or treatment) and (malaria*))) or

ab=(antimalarial* or anti-malarial* or ((schizonticidal* or gametocidal* or hypnozoiticidal* or drug* or treatment) and (malaria*))) or

de=“antimalarials”

B. Mass Administration

(mass) and (administ* or distribut* or applicat*)

LILACS

A. Anti-Malarials

antimalarial* or anti-malarial* or ((schizonticidal* or gametocidal* or hypnozoiticidal* or drug* or treatment) and (malaria*))
