# Supplementary material for: Community engagement and population coverage in mass anti-malarial administrations: a systematic literature review
Source: Malar J. 2016 Nov 2;15:523. doi: 10.1186/s12936-016-1593-y (PMC5093999; doi:10.1186/s12936-016-1593-y)
Supplement: Supplementary file 2 — Additional file 2. Studies that did not document either community engagement or population coverage (n = 47). [file 12936_2016_1593_MOESM2_ESM.docx]

**Additional file 2: Studies that did not document either CE or Population Coverage (n=47)**

| **Author, Year** | **Country** | **Drug** |
| --- | --- | --- |
| Aliev 2000 | Tajikistan | Primaquine |
| Parrot 1937 | Algeria | Q, Quinacrine + Proequine |
| Van Goor 1950(a) | Tjiandjur, Indonesia | Proguanil |
| Van Goor 1950(b) | Tjiandjur, Indonesia | Proguanil |
| Abraham 1944 | India | Quinine |
| Allen 1990 | The Gambia | Pyrimethamine + Dapsone |
| Amangel'diev 2001 | Turkmenistan | Dellaguil |
| Annual Report 1932 | Malaysia | Quinine + Plasmoquine |
| Barber 1932 | Liberia | Plasmoquine |
| Berberian 1948b | Lebanon | Chloroquine |
| Bloch 1982 | El Salvador | Unspecified 4-aminoquinoline |
| Brink 1958 | Transvaal (South Africa) | Chloroquine, pyrimethamine |
| Chaudhuri 1950b | India | Proguanil, Amodiaquine, Chloroquine |
| Clark 1942 | Panama | Quinine + Plasmochine, Mepacrine + Plasmochine |
| Clyde 1961b | Tanzania | Quinine |
| De Zulueta 1961 | Uganda | Chloroquine + Pyrimethamine |
| De Zulueta 1964 | Uganda | Chloroquine + Pyrimethamine |
| Desowitz 1987 | Papua New Guinea | Chloroquine |
| Dixon 1950 | Kenya | Proguanil |
| Doi 1989a | Indonesia | SP + Primaquine |
| Gilroy 1952 | India | Proguanil, Chloroquine |
| Han 2006 | Republic of Korea | Chloroquine + Primaquine |
| Harwin 1973 | Rhodesia (Zimbabwe) | Cycloguanil + Chloroquine |
| Jones 1958 | Kenya | Pyrimethamine |
| Klopper 1949 | Netherlands | Proguanil |
| Komp 1935 | Panama | Quinine, Mepacrine, Plasmochin, Mepacrine + Plasmochin |
| Laing 1970a | The Gambia | SP, Pyrimethamine |
| Laing 1970b | The Gambia | SP, Pyrimethamine |
| Lysenko 1960a | USSR | Quinocide |
| Lysenko 1960b | USSR | Quinocide |
| Mackerras 1954 | Australia (Torres Straits Islands) | Mepacrine + Pyrimethamine |
| Malaria_Army 1934 | India | Plasmoquine |
| Mason 1973 | El Salvador | Pyr+PQ |
| Mason 1977 | El Salvador | AQ+PQ, Pyr+PQ |
| Mastbaum 1957 | Swaziland | Pyrimethamine |
| Nave 1973 | El Salvador | Chloroquine + Primaquine |
| Peters 1962 | New Guinea, Solomon Islands, New Hebrides | Pyr, Pyr+CQ |
| Rachoux 1965 | El Salvador | Chloroquine, primaquine |
| Rafi 1951 | Pakistan | Paludrine |
| Saarinen 1987 | Angola | Chloroquine, Proguanil |
| Seckinger 1935 | Georgia, US | Atebrine, plasmochin |
| Singh 1953 | India | Amodiaquine |
| Srivastava 1950 | Tarai (Nepal) | Paludrine |
| Strangeways 1950 | Kenya | Paludrine |
| Villegas 2010 | Suriname | ND |
| Wallace 1936 | Malaysia | Mepacrine + Plasmochin, Quinoplasmine |
| Wallace 1954 | Malaysia | Proguanil, Chloroquine |
